# Rupture of Extensor Pollicis Longus Tendon Caused by Scaphoid Nonunion Advanced Collapse Wrist

**DOI:** 10.1155/2020/8850427

**Published:** 2020-08-10

**Authors:** Takahito Kojima, Masataka Yasuda, Shunpei Hama, Makoto Fukuda, Kenta Minato

**Affiliations:** Department of Orthopaedic Surgery, Baba Memorial Hospital, Osaka, Japan

## Abstract

We report the case of a 69-year-old male patient with extensor pollicis longus (EPL) tendon rupture associated with a scaphoid nonunion advanced collapse (SNAC) wrist. He could not actively extend the left thumb interphalangeal joint and visited our institution for an examination. Plain X-rays revealed advanced stage SNAC and an enlarged soft tissue shadow owing to dorsal ridge growth. The patient was diagnosed with EPL tendon subcutaneous rupture due to SNAC. During surgery, the EPL tendon was found to be absent, a proximal-type scaphoid nonunion was detected, and bone growth to the dorsal part of the dorsal ridge was observed. Considering that the EPL tendon rupture was associated with the bone growth, we performed scaphoid lunate advanced collapse (SLAC) reconstruction and extensor indicis proprius tendon transfer which needed a revision tendon surgery afterward. To the best of our knowledge, EPL tendon ruptures caused by SLAC or SNAC are considered rare and have not yet been reported.

## 1. Introduction

Rupture of the extensor pollicis longus (EPL) tendon may result from fractures, surgery, trauma, rheumatoid arthritis, or corticosteroid use. We report the case of a patient with an EPL tendon rupture associated with a scaphoid nonunion advanced collapse (SNAC) wrist [1] and a literature review.

## 2. Case Presentation

The patient, a 69-year-old man, could not actively extend the left thumb interphalangeal (IP) joint approximately 10 days before presentation and was referred to our institution for an examination. The patient had experienced pain in the wrist joint since a long period. However, the patient did not participate in sports and had no recollection of any trauma and infection. His medical history included pyogenic spondylitis and autoimmune hepatitis, and he had been undergoing corticosteroid therapy (5 mg) for 1 year. Although inability of active extension of the left thumb and swelling in the snuff box were observed, there was no tenderness. Grip strength was 25 kg in the right hand and 11 kg in the left hand, right wrist extension and flexion were 65° each, and left wrist extension and flexion were 60° and 50°, respectively.

**Figure 1:**
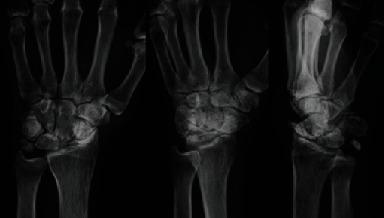
Plain X-ray findings show sharpening of the styloid process, narrowing between the radial scaphoid bones, and growth of the dorsal ridge.

Plain X-rays revealed advanced stage SNAC in the left hand; supinated oblique view revealed an enlarged soft tissue shadow due to the growth of the dorsal ridge (Figure 1). We diagnosed the patient with EPL tendon subcutaneous rupture due to SNAC.

**Figure 2:**
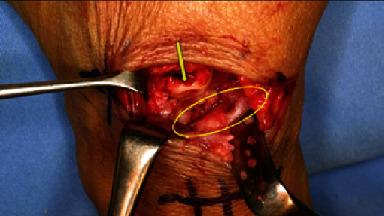
A cartilage defect is observed in the capitate bone (arrow). A proximal-type scaphoid pseudarthrosis with bony growth into the dorsal ridge is observed (oval).

**Figure 3:**
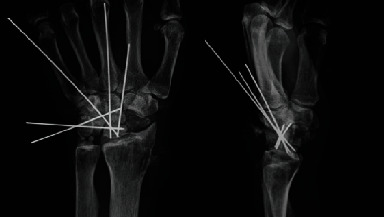
X-ray image obtained immediately after the surgery using the Watson method.

We performed the surgery 10 weeks after the initial diagnosis, because the patient's general condition was not favorable due to pyogenic spondylitis. During the surgery, when the dorsal part of the wrist was opened, the EPL tendon was found to be absent. We noted scarring surrounding the snuff box, perforation of the joint capsule, and a cartilage defect in the capitate head. A proximal-type scaphoid nonunion was detected, and bone growth to the dorsal part of the dorsal ridge was observed (Figure 2). The EPL tendon rupture was attributed to the bone growth. We performed scaphoid lunate advanced collapse (SLAC) reconstruction using a 4-corner fusion procedure described by Watson and Ballet [1], replacing the missing EPL tendon with the extensor indicis proprius (EIP) tendon using Pulvertaft weave fashion (Figure 3). A long thumb spica cast was used for the patient until week 3 following surgery, and a short thumb spica cast was used from week 4 to week 7. The cast was removed 7 weeks after surgery; however, active extension of the thumb again became impossible.

**Figure 4:**
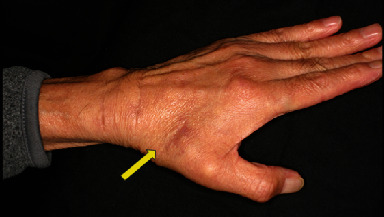
Postoperative macroscopic findings. Improvements in the EPL tendon could not be confirmed, and the transferred tendon is not palpable in the snuff box (arrow).

**Figure 5:**
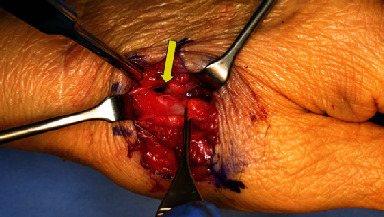
Macroscopic findings at the time of the second surgery. EPL tendon rerupture is shown to have occurred in the dead space where the scaphoid bone was previously located.

At 6 months after surgery, extension and flexion in the left wrist were 30° and 35°, respectively. The patient was unable to extend his thumb, improvement in the transferred tendon function could not be confirmed, and the transferred tendon was not palpable in the snuff box (Figure 4). Accordingly, a tendon transfer using the palmaris longus (PL) tendon was planned based on a diagnosis of transferred EIP tendon rerupture. The transferred EIP tendon had reruptured in the dead space where the scaphoid bone was previously located (Figure 5). The PL tendon and distal palmar aponeurosis were taken, according to the Camitz method [2], and transferred to the EPL tendon using Pulvertaft weave fashion. A thumb spica cast was applied for 3 weeks. Although active extension of the left thumb was possible 1 year after the second surgery, a 20° extension lag persisted. Left hand grip strength was 21 kg, left thumb IP joint extension was 5°, and flexion was 75°. Disabilities of the Arm, Shoulder and Hand score was 29.3 points.

## 3. Discussion

EPL tendon rupture is caused by mechanical irritation due to Lister's tubercle and rheumatoid arthritis. Björkman and Jörgsholm [3] retrospectively investigated and reported the cause of EPL tendon ruptures in 27 patients, of which 14 patients reported distal radius fractures, surgical pin fixation, or plate fixation; 5 reported blunt trauma; 6 reported corticosteroid use for systemic disease; and 2 reported receiving corticosteroid injections. Zinger et al. [4] described the case of a patient in whom a fracture caused by a deeper-than-usual bone morphology in the third compartment of Lister's tubercle resulted in spontaneous EPL tendon rupture without the aforementioned risk factors. Although the risk factors were not involved in that patient, the EPL tendon rupture was attributed to excessive and repetitive use of the wrists at work and in sports. However, in the present patient, no anatomical abnormalities or an excessive use of the hand was noted. We considered bone proliferation due to arthropathic changes to be the cause of the EPL tendon rupture.

FJ Harvey and PM Harvey [5] described a patient with extensor digitorum communis and EIP tendon rupture due to bone fragments associated with scaphoid nonunion. There have been no reports of extensor digitorum communis (EDC) tendon rupture due to scaphoid nonunion for 30 years before that report, suggesting that EDC tendon rupture due to a scaphoid nonunion or SNAC is rare. Furthermore, Zachee et al. [6] reported the case of a patient with flexor pollicis longus (FPL) tendon injury due to scaphoid nonunion. X-ray findings showed a SNAC wrist and an intraoperative degenerative FPL tendon rupture at the level of the tubercle of the scaphoid bone. They reported that tendon rupture at the wrist is rare, with only 4 cases reported previously.

In the present patient, a SNAC wrist due to scaphoid nonunion was observed, which we believe caused the EPL tendon rupture. Although the patient reported corticosteroid use, it was unlikely that this was the cause of the tendon rupture owing to the low corticosteroid dose; however, the possibility of an effect could not be ruled out.

Although SLAC reconstruction was performed as a therapeutic approach, the transferred tendon rerupture was observed. Because the transferred tendon rerupture occurred in the dead space where the scaphoid bone was resected, ischemia was considered the cause. Although the surgery was performed by an experienced surgeon, the possibility of technical errors occurring during surgery cannot be ruled out.

To the best of our knowledge, EPL tendon ruptures caused by SLAC or SNAC are considered rare and have not yet been reported.
